# Spin-Scaled Range-Separated Double-Hybrid Density Functional Theory for Excited States

**DOI:** 10.1021/acs.jctc.1c00422

**Published:** 2021-06-21

**Authors:** Dávid Mester, Mihály Kállay

**Affiliations:** Department of Physical Chemistry and Materials Science, Budapest University of Technology and Economics, P.O. Box 91, H-1521 Budapest, Hungary

## Abstract

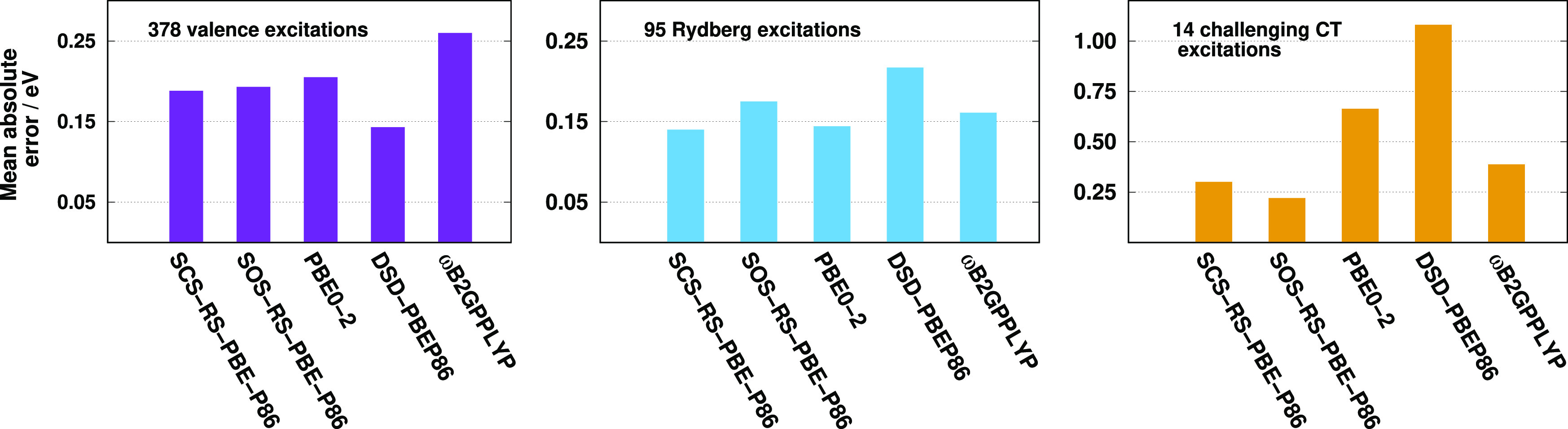

Our recently presented range-separated (RS) double-hybrid (DH) time-dependent density functional approach [*J. Chem. Theory Comput.***17,** 927 (2021)] is combined with spin-scaling techniques. The proposed spin-component-scaled (SCS) and scaled-opposite-spin (SOS) variants are thoroughly tested for almost 500 excitations including the most challenging types. This comprehensive study provides useful information not only about the new approaches but also about the most prominent methods in the DH class. The benchmark calculations confirm the robustness of the RS-DH ansatz, while several tendencies and deficiencies are pointed out for the existing functionals. Our results show that the SCS variant consistently improves the results, while the SOS variant preserves the benefits of the original RS-DH method reducing its computational expenses. It is also demonstrated that, besides our approaches, only the nonempirical functionals provide balanced performance for general applications, while particular methods are only suggested for certain types of excitations.

## 1 Introduction

Nowadays, the density functional theory (DFT) is one of the most popular tools in quantum chemistry, although it is still challenging to select the most suitable density functional approximations for particular purposes. Numerous comprehensive benchmark studies are available for ground-state calculations^1−5^ that provide an opportunity for some insights into the performance of the methods. However, the availability of such studies for excited-state properties is rather limited, considering especially the most recent DFT approaches. On the one hand, only a limited number of high-level, extensive, and reliable benchmark sets were available previously. On the other hand, the best approaches have been just published in recent years.

Currently, the time-dependent DFT (TDDFT)^6−10^ is the method of choice for excited states of extended molecular systems since its computational costs are relatively low. However, it is well-known that the reliability of the results is frequently in question. The TDDFT methods using pure exchange–correlation (XC) functionals are highly not recommended, while hybrid functionals, where the XC energy contains Hartree–Fock (HF) exchange contribution as well, can still fail for challenging cases.^11−14^ This drawback can originate from the wrong long-range (LR) behavior of the standard XC functionals and causes significant problems for Rydberg and charge transfer (CT) states or π → π* excitations of conjugated systems. Thus, numerous approaches, such as the range-separated (RS) and double-hybrid (DH) theories were developed to enable their general usage for both the ground- and excited-state properties.

The separation of the Coulomb interaction into LR and short-range (SR) components was proposed by Savin and co-workers.^15,16^ In their functionals, the LR (SR) part of the exchange energy is dominantly covered by the LR HF (SR DFT) energy, while the DFT correlation contribution remains unaltered. It has been demonstrated in several studies that such methods, for example, the LC-BOP,^17−20^ CAM-B3LYP,^21^ and ωB97^22^ approaches, are significantly superior to the standard hybrid methods.^1,2,23−27^ Another widely used technique to improve the results is the DH theory introduced by Grimme.^28^ In this case, the hybrid Kohn–Sham (KS) energy is augmented with a second-order Møller–Plesset (MP2)-like correction evaluated on the orbitals obtained. The parameterization of the first DHs was based on empirical considerations,^28−30^ while nonempirical approaches^31−36^ were later derived from the adiabatic connection formalism. Spin-scaled DH variants^37−44^ were also proposed, where the perturbative correction is replaced by the spin-component-scaled (SCS)^45^ or scaled-opposite-spin (SOS)^46^ MP2 correction. It was shown that, in general, empirical functionals provide more reliable results for ground-state properties compared to the nonempirical ones, while the spin-scaled DHs are the clear superiors.^1−5,38^ The first attempts to utilize the RS and DH approaches together were made by Ángyán, Toulouse, and Savin et al.,^47,48^ while the necessary technicalities were elaborated by Toulouse,^49,50^ Stoll,^51−53^ and their co-workers. Later, several RS-DH approaches were proposed where the LR correlation energy is evaluated at the MP2 level^54,55^ or beyond.^56−59^ The more approximate form of the theory, the family of the so-called LR-corrected functionals, is also noteworthy, where solely, the exchange contributions are range-separated.^37,60−63^

The above approaches were extended to excited-state calculations as well. The generalization of the RS hybrid functionals is fairly straightforward, whereas the basics of the excited-state DH theory were elaborated by Grimme and Neese.^64^ In their approach, a hybrid TDDFT calculation is performed, and subsequently, the effect of double excitations is added *a posteriori* relying on the configuration interaction singles with the perturbative second-order correction [CIS(D)]^65^ method. We note that the second-order algebraic-diagrammatic construction method^66^ can also be considered as a natural excited-state extension of the MP2 method. Thus, an excited-state DH analogue can be also defined relying on it.^67^ The first spin-scaled variants of the genuine ansatz were considered by Schwabe and Goerigk,^68^ while the first LR-corrected DHs were also proposed by the same group.^69^ Our RS-DH functionals,^70^ where both the exchange and correlation contributions are range-separated, were recently published.

These methodological developments significantly improve the performance of the functionals for excited states, however, only a few comprehensive studies^71,72^ can be found on the comparison of the most recent methods. In addition, the widely used compilations, which were regularly used in the earlier studies, contain low-level references,^73−76^ only certain type of excitations,^77−79^ or they are simply not challenging enough. These problems were recently resolved by several authors. One of the most promising attempts is the QUEST database created by Loos, Jacquemin, and co-workers,^80^ which contains different types of benchmark compilations and high-level reference energies. The updated reference values computed by Goerigk et al.^68,72^ for the well-balanced Gordon test set^75^ are also noteworthy, while a challenging CT benchmark set was compiled by Szalay and co-workers.^81^ In all cases, the high-level reference values were calculated at the coupled-cluster (CC) level including triple excitation correction, such as the CC3,^82^ the CCSDR(3),^83^ and the CCSDT-3^84^ approaches. The simultaneous usage of these benchmark compilations enables the comprehensive assessment of the most recent functionals.

In this paper, we combine our recent RS-DH ansatz^70^ with spin-scaling techniques for excited-state calculations. After a brief review of the theory, we determine the mixing factors of the corresponding contributions, and then, we demonstrate the robustness of our ansatz through numerous benchmark calculations using the aforementioned benchmark sets. The performance of the new methods is compared to that of the most accurate DH functionals, as well as the CC singles and doubles (CCSD)^85^ approach and its approximate second-order form (CC2)^86^ are also assessed.

## 2 Theory and Methodology

### 2.1 Spin-Scaled Ansatz for the Genuine DHs

The ground-state XC energy in the DH theory^28^ is calculated as
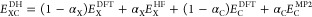
1where *E*_X_^DFT^ and *E*_C_^DFT^ are the semilocal exchange and correlation energies, respectively, while *E*_X_^HF^ denotes the exact (HF) exchange energy, and *E*_C_^MP2^ is the MP2 correlation energy. α_X_ and α_C_ stand for the mixing factors of the HF and MP2 contributions, respectively. In this two-step scheme, a calculation starts with solving the hybrid KS equations including the DFT exchange and correlation potentials, as well as the HF exchange contribution, and the energy is augmented with the MP2 contribution evaluated on the KS orbitals. The most widely used excited-state extensions of DFT are TDDFT^6^ and its simplified version, the so-called Tamm–Dancoff approximation (TDA),^87^ whereas the CIS(D)^65^ approach can be considered as one of the corresponding excited-state analogues of the MP2 method. Numerous benchmark studies have shown that significant differences cannot be observed between the TDA- and “full” TDDFT approaches for singlet excitation energies,^72,76,87^ while only TDA-TDDFT is recommended for triplet transitions due to the triplet instability of TDDFT.^10,72,88^ Accordingly, one of the simplest schemes is utilized here, relying on TDA-TDDFT and CIS(D). In this manner, the first step of a calculation is to solve the hybrid TDA-TDDFT eigenvalue equation as
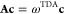
2where **A** is the Jacobian matrix, **c** is the singles excitation vector, and ω^TDA^ stands for the TDA excitation energy. Thereafter, the second-order correction is calculated, perturbatively relying on the CIS(D) method using the single excitation amplitudes and excitation energy obtained from the previous equation. The final DH excitation energy^64^ is defined by
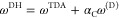
3where ω^(D)^ is the perturbative second-order correction. The effective implementation of DHs and the corresponding working equations were previously discussed in detail in refs (64) and (70).

In the more complicated SCS DHs,^41^ the opposite-spin (OS) and same-spin (SS) contributions to the MP2 correlation energy, *E*_C_^OS-MP2^ and *E*_C_^SS-MP2^, are scaled separately by factors α_C_^OS^ and α_C_^SS^, respectively, as
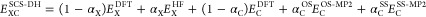
4

In this case, one has four adjustable parameters in the XC energy in contrast to the genuine DHs, where only two parameters can be varied. Accordingly, this ansatz enables higher flexibility of the energy functional and ensures more accurate description of the chemical properties.^2,42−44^ In the case of the SOS variant, the number of the adjustable parameters is reduced to three as the SS contributions are completely neglected. It is important to note that the computational scaling of *E*_C_^OS-MP2^ can be reduced to *N*^4^, invoking the density fitting approximation for the two-electron integrals and a Laplace-transform-based trick,^46^ whereas the scaling of the *E*_C_^SS-MP2^ is *N*^5^, where N is a measure of the system size. The spin-scaling techniques can also be utilized for excited-state calculations,^68^ where the final excitation energy is obtained as
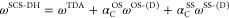
5with ω^OS-(D)^ and ω^SS-(D)^ as the OS and SS contributions to the second-order correction, respectively. The SOS variant can also be defined in this case, and the computational requirements can be reduced similarly to the ground-state calculations.^89^

### 2.2 Spin-Scaled Ansatz for the Two-Parameter RS DHs

A two-parameter RS-DH ansatz utilizing the Coulomb-attenuating method (CAM)-like decomposition^21^ of the Coulomb potential was proposed by Kalai and Toulouse,^54^ where both the exchange and correlation contributions are range-separated. In this scheme, the XC energy is defined by
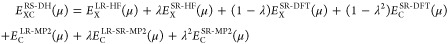
6where *E*_X_^SR-DFT^ and *E*_C_^SR-DFT^ stand for the SR DFT exchange and correlation contributions, respectively, and *E*_X_^SR-HF^ denotes the SR HF exchange, while *E*_C_^SR-MP2^ is the SR MP2 correlation energy. A similar notation is applied to the LR analogues of the latter two terms. The mixed LR-SR contribution, *E*_C_^LR-SR-MP2^, also appears in the expression since the MP2 energy is nonlinear in the Coulomb potential. The parameter λ can be interpreted as the weight of the wave function methods in the XC energy, while μ stands for the range-separation parameter. To evaluate the SR DFT contributions, the local-scaling approximation of Scuseria and co-workers has been adapted.^59,70^ Notice that well-defined energy formulas are retrieved in the limits of the λ and μ parameters. First, the approach simplifies to the standard KS-DFT if one sets μ = 0 and λ = 0. In the μ → ∞ or λ = 1 limits, the standard MP2 method is recovered. In addition, a genuine DH-like approach is recovered for μ = 0 and 0 < λ < 1, while the one-parameter RS ansatz introduced by Ángyán et al.^47^ is obtained in the 0 < μ < ∞ and λ = 0 case. Relying on the two-parameter RS-DH approach, we have recently proposed the
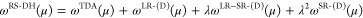
7expression for the excitation energies,^70^ where ω^LR-(D)^, ω^SR-(D)^, and ω^LR–SR-(D)^ denote the LR, SR, and mixed contributions to the second-order correction, respectively. The working equations and the advantages of the ansatz compared to the LR-corrected DH functionals^69,72^ are presented in detail in ref (70).

The spin-scaling techniques can also be combined with the above ansatz to merge the benefits of the two schemes. It is trivial that the RS MP2 contributions can be split into OS and SS terms as well. Accordingly, in the final energy expression, the contributions are simply scaled by the corresponding mixing factors as
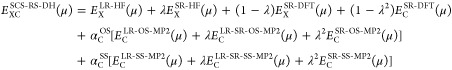
8

In this case, one has four variable parameters similarly to the genuine spin-scaled DH ansatz, and the SOS variant can also be defined with three parameters. Akin to eq 6, the observations regarding the limits of the λ and μ parameters are still preserved. In addition, the two-parameter RS-DH ansatz is recovered if one sets α_C_^OS^ = 1.0 and α_C_^SS^ = 1.0. The analogous expression that we propose for the excitation energies reads as
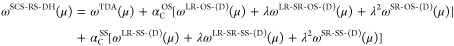
9

The working equations needed for implementing eqs 8 and 9 are practically identical to those required for eqs 6 and 7. Accordingly, an existing code can be used without any modification if it is suitable for spin scaling.

### 2.3 Computational Details

The new approach has been implemented in the MRCC suite of quantum chemical programs and will be available in the next release of the package.^90,91^

Several basis sets were used in this contribution, such as Dunning’s correlation consistent basis sets (cc-pVXZ, where X = D and T),^92,93^ their diffuse function augmented variants (aug-cc-pVXZ),^94^ as well as Ahlrichs’ TZVP^95^ basis sets. In the calculations, the density-fitting approximation was invoked for both the ground and the excited states. For this purpose, the corresponding auxiliary bases of Weigend et al.^96−98^ were employed. At all the figures or tables, the corresponding basis sets are specified. The frozen core approximation was utilized in all the post-KS/HF steps.

The most successful DH functionals, namely, the empirical B2GPPLYP,^30^ its LR-corrected variant ωB2GPPLYP,^69^ the nonempirical PBE0-2 and^34^ PBE-QIDH,^36^ as well as the spin-component-scaled DSD-PBEP86 were selected for comparison with our ansatz. Their accuracy for ground-state properties is well-documented in the literature through excellent benchmark studies.^1−5^ However, less is known about their performance for excited-state calculations since fewer comprehensive studies have been published on this issue. On the basis of the available results, it can be stated that global DH functionals systematically outperform global hybrid ones,^71,73,74,99,100^ and the accuracy of RS hybrids is far from the best DHs in general.^70^ Within the TDA approximation, the best performer empirical standard DH is the B2GPPLYP.^68,72^ Surprisingly, the nonempirical PBE0-2 functional shows a more balanced performance compared to the former one,^68,70^ while the workhorse spin-scaled variant is the DSD-PBEP86.^68^ The LR-corrected ωB2GPPLYP approach enables the significantly better description of the Rydberg and CT excitations; however, it is less advantageous for valence transitions compared to the B2GPPLYP functional.^70,72^ As it was pointed out in ref (72), for excited-state calculations, ωB2GPPLYP is clearly superior to the LR-corrected PBE-based nonempirical functionals, such as RSX-QIDH.^60−62^ Our recent two-parameter RS-DH approaches^70^ can be considered as one of the most robust and accurate alternatives within the DH theory. As it was shown in ref (70), the same parameter set was optimal for all the presented combinations of exchange and correlation functionals and their performances were very similar. Accordingly, for the sake of simplicity, only the RS-PBE-P86 and its spin-scaled variants are assessed in this paper. The collection of these functionals will be hereafter referred to as RS DHs, while the ωB2GPPLYP functional will be referenced as LR-corrected DH.

To calculate the DFT contributions, Becke’s 1988 exchange functional (B88),^101^ the correlation functional of Lee, Yang, and Parr (LYP),^102^ the exchange and correlation functionals of Perdew, Burke, and Ernzerhof (PBE),^103^ as well as Perdew’s 1986 correlation functional (P86)^104^ were applied. To obtain the SR DFT contributions utilizing the local-scaling approximation,^59^ the Slater–Dirac exchange^105−107^ and the Perdew–Wang 1992 correlation^108^ functionals were used as local-density approximation functionals together with their SR extensions proposed by Savin^109^ and Paziani et al.^110^ The built-in functionals of the MRCC package were used in all cases, except for the ωB2GPPLYP calculations, where the modified version of the Libxc library^111,112^ was employed. To help the reader, the attributes of the functionals assessed in our study are presented in Table 1.

**Table 1:** DH Functionals Assessed in This Paper

functional	exchange	correlation	range separation	spin scaling
DSD-PBEP86	PBE	P86	no	yes
RS-PBE-P86	PBE	P86	yes	no
SCS-RS-PBE-P86	PBE	P86	yes	yes
SOS-RS-PBE-P86	PBE	P86	yes	yes
B2GPPLYP	B88	LYP	no	no
ωB2GPPLYP	B88	LYP	yes	no
PBE0-2	PBE	PBE	no	no
PBE-QIDH	PBE	PBE	no	no

### 2.4 Benchmark Sets

In order to retain the consistency with the previous DH studies,^67−70,72^ our training and validation sets were selected from the literature. For most of them, singlet and triplet excitations are also available. The spin-scaling parameters were tuned for the singlet excitations of the well-balanced benchmark set of Gordon and co-workers,^75^ which includes 32 valence and 31 Rydberg transitions. The updated reference energies were obtained at the composite CC3-CCSDR(3)/aug-cc-pVTZ approach by Schwabe and Goerigk (SG)^68^ using reoptimized high-level geometries. The updated triplet transitions were recently published by Casanova-Páez and Goerigk.^72^ This compilation is less balanced and contains 28 valence and 10 Rydberg excitations obtained at the same level as the singlet ones. Cross-validation has been performed on several popular test sets. One of the most widely used benchmark sets was proposed by Thiel and co-workers.^77,78^ This set is a compilation of CC3/TZVP reference values, and 121 singlet and 71 triplet excitations were selected. This test set only incorporates valence excitations. The singlet transitions were later reconsidered by Kánnár and Szalay,^113^ and these results were used as a reference in this study. Two different benchmark compilations were presented by Loos, Jacquemin, and co-workers.^114,115^ Their first set (LJ1)^114^ contains 55 singlet (29 Rydberg and 26 valence) and 47 triplet (18 Rydberg and 29 valence) excited states of small organic molecules, and CC3/aug-cc-pVTZ excitation energies were used as a reference. Their second benchmark set (LJ2)^115^ consists of 19 singlet and 11 triplet excitation energies of so-called “exotic” molecules evaluated at the same level.

Some of the benchmark sets contain only singlet excitations, such as the training set of SG,^68^ which is hereafter referred to as the SG set. This compilation consists of CCSDR(3) excitation energies using the aug-cc-pVTZ basis set and includes 38 excitation energies (32 valence and 6 Rydberg) for 22 molecules. The comprehensive CT benchmark set recently proposed by Szalay et al.^81^ contains 14 excitation energies evaluated at the CCSDT-3 level using the cc-pVDZ basis sets. This set comprises eight molecular complexes at a large distance, such as ammonia–fluorine, acetone–fluorine, pyrazine–fluorine, ammonia–oxygen-difluoride, acetone–nitromethane, ammonia–pyrazine, pyrrole–pyrazine, and tetrafluoroethylene–ethylene systems, to ensure the high CT character of the transitions. In addition, a test set on the ^1^L_a_ and ^1^L_b_ excitations of the linear polycyclic aromatic hydrocarbons (PAHs) from naphthalene to hexacene is also discussed. The reference values^79^ were obtained at the completely renormalized equation-of-motion CCSD(T) [CR-EOM-CCSD(T)]^116^ level using the cc-pVTZ basis set. All in all, 320 singlet and 167 triplet excitations are inspected in this study. Taking into account the wide range of phenomena studied, we can state that this work can be considered as one of the most comprehensive studies in this field.

The main statistical error measures presented in the tables and figures are the mean error (ME), the mean absolute error (MAE), and the maximum absolute error (MAX). The errors utilized for the evaluation of the excitation energies are calculated by subtracting the reference from the computed value. All the computed excitation energies and statistical error measures are available in the Supporting Information. In addition, further measures, such as the root mean square error (RMSE), standard deviation (SD), and deviation span are also included. These numbers are only discussed if the order of the methods significantly changes when evaluating their performance using the latter measures instead of the former ones.

## 3 Results

### 3.1 Determination of the Spin-Scaling Parameters

**Figure 1:**
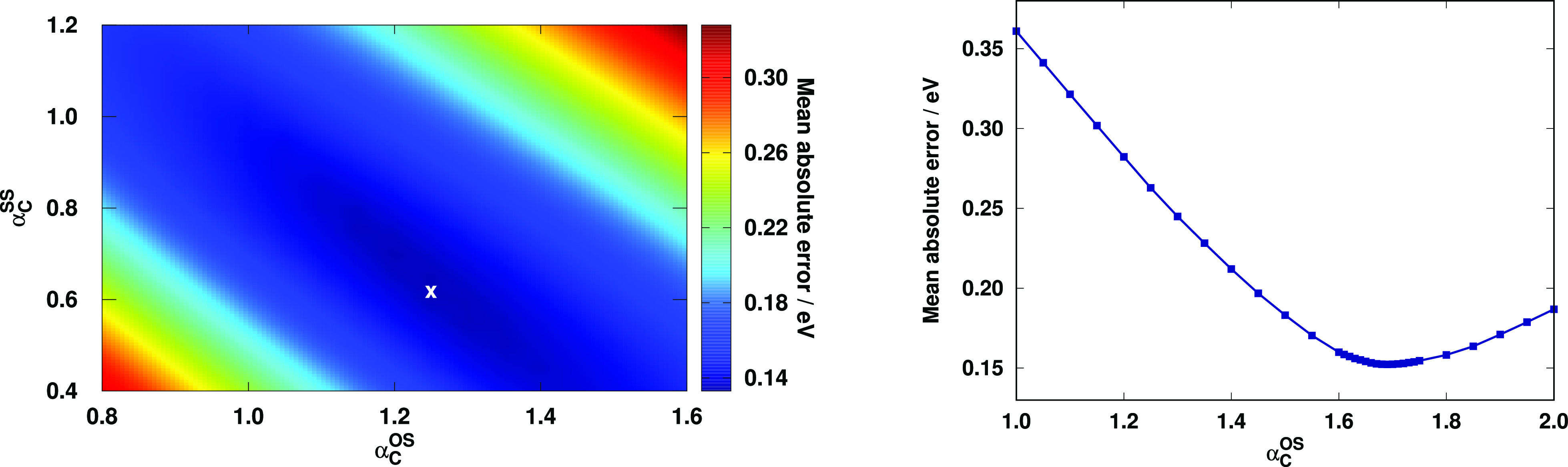
MAEs for the singlet excitations of the Gordon test set^72,75^ for the SCS (left panel) and SOS (right panel) variants using the aug-cc-pVTZ basis set with the corresponding auxiliary bases. In the case of the SCS variant, the white X marks the global minimum.

First, we determined the optimal spin-scaling parameters using the singlet excitations of the well-balanced Gordon test set. For this purpose, the α_C_^OS^ and α_C_^SS^ values were scanned, and the MAE for the SCS and SOS variants was minimized. To preserve compatibility with our RS-DHs, the default parameters of λ = 0.7 and μ = 0.5 bohr^–1^ obtained for the same training set in ref (70) were retained. The results are presented in Figure 1. Foremost, we discuss the SCS variant in detail. Concerning the unscaled ansatz as a starting point, the MAE slowly decreases with decreasing α_C_^SS^ and increasing α_C_^OS^ parameters. The global minimum can be found at α_C_^OS^ = 1.24 and α_C_^SS^ = 0.64, while the MAE is 0.13 eV at this point. The minimum is fairly shallow and several optimal pairs can be determined in the ranges of 0.4 ≤ α_C_^SS^ ≤ 0.7 and 1.1 ≤ α_C_^OS^ ≤ 1.4. It means that the lower weight of one of the contributions can be compensated by the higher weight of the other ones. In the case of the SOS variant, the global minimum is well-defined and can be found at α_C_^OS^ = 1.69. The error decreases rapidly until α_C_^OS^ = 1.60, while it increases slowly after the minimum. The lowest MAE is 0.15 eV, which is higher only by 0.02 eV compared to the SCS variant. We note that the reoptimization of the parameters, including the λ and μ parameters as well, has only a negligible effect on the results.

**Figure 2:**
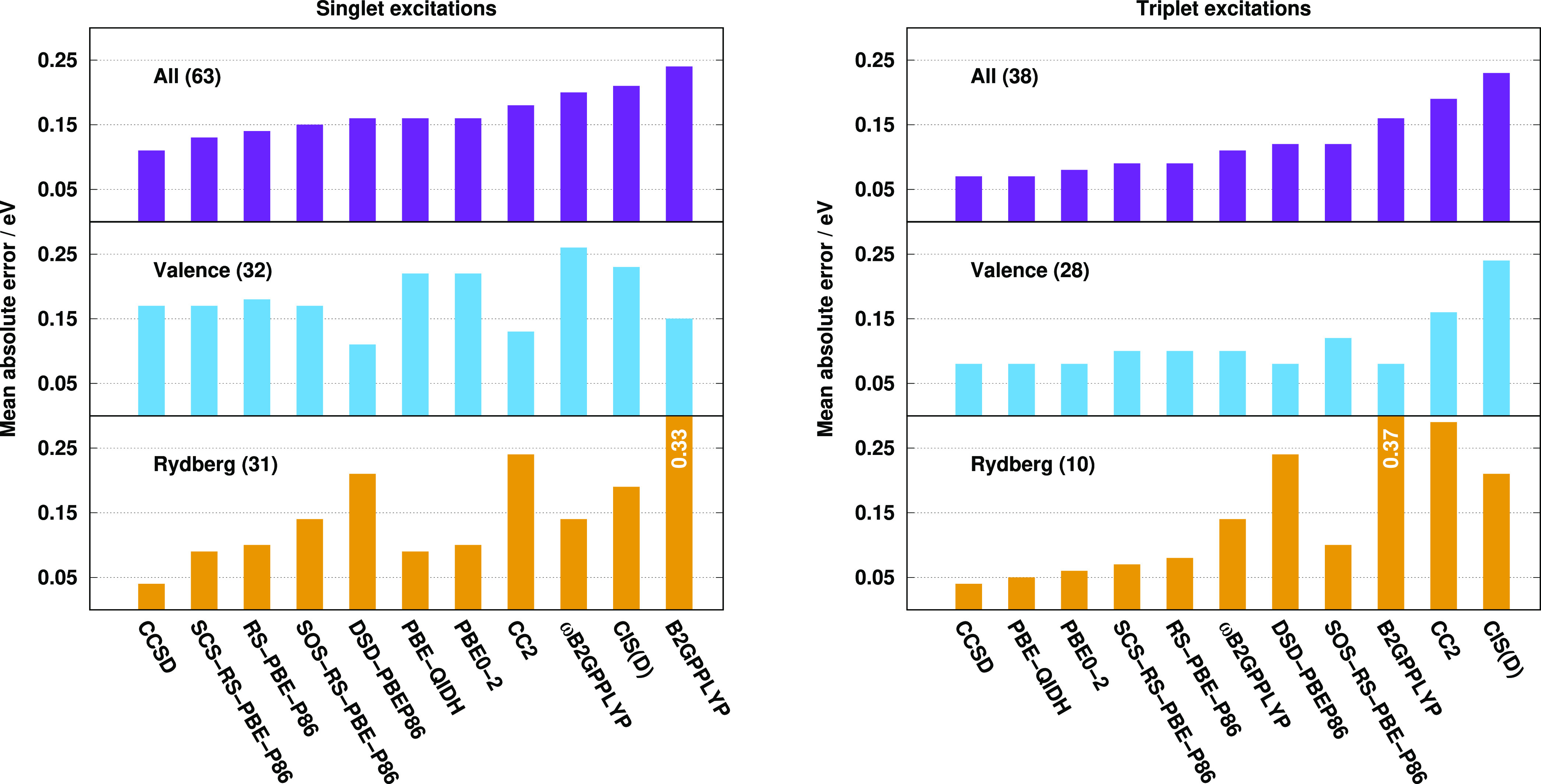
MAEs for the calculated singlet (left panel) and triplet (right panel) excitation energies for the Gordon test set^72,75^ using the aug-cc-pVTZ basis sets with the corresponding auxiliary bases. The number of the transitions can be found in parentheses. The B2GPPLYP, ωB2GPPLYP, PBE-QIDH, and CCSD values were taken from ref (72).

Thereafter, we compare the performance of the spin-scaled RS-DH approaches for the Gordon test set to other methods. For this purpose, the MAEs with the default parameters for various types of excitations were assessed. The results are visualized in Figure 2. Inspecting the bars for the singlet excitations, we can observe that the best performance is attained by the CCSD method, to which the overall MAE is 0.11 eV. The error is still below 0.15 eV for all the RS-DH approaches. It is 0.16 eV for the DSD-PBEP86 and PBE-based functionals, while the MAE starts to increase for the remaining methods. The valence excitations are rather well-balanced for the best approaches, and the best performer is the DSD-PBEP86 functional. The spin-scaling slightly improves the results for the RS DHs for both variants. For the Rydberg states, the most outstanding methods are the CCSD, (SCS-)RS-PBE-P86, and the PBE-based approaches. Unfortunately, the lack of the SS contribution increases the error in the case of SOS-RS-PBE-P86; however, it is still more reliable than the remaining approaches.

It is interesting to see that the overall MAEs are roughly halved for the triplet excitations concerning the best methods. Besides the CCSD method, the PBE-QIDH and PBE0-2 functionals are the superiors, while the (SCS-)RS-PBE-P86 functionals are also outstanding with MAEs of 0.09 eV. The worst results were obtained by the fifth-order scaling wave-function-based methods. The valence excitations are well-balanced, and salient functionals cannot be identified. In this regard, the MAEs with all the functionals are highly acceptable, and the largest deviation between the MAEs is only 0.04 eV. The error is somewhat higher for SOS-RS-PBE-P86 compared to SCS-RS-PBE-P86. The MAEs for the Rydberg excitations are less consistent. For most of the functionals, it is below 0.10 eV, whereas the error is 0.14 and 0.24 eV for the ωB2GPPLYP and DSD-PBEP86 approaches, respectively. The errors for the Rydberg excitations are consistently lower compared to the singlet results, except for the aforementioned functionals, while the description of the valence excitations is significantly better with all the TDA-TDDFT methods.

The compilation of additional statistical error measures for the Gordon set can be found in Table 2. The lowest MEs can be achieved through significant error cancellation for the singlet excitations. As it can be seen, the ME has an opposite sign for the valence and Rydberg transitions for the best performers, such as CIS(D) and (SCS-)RS-PBE-P86. We note that the lowest SDs and RMSEs were provided by the CCSD and the RS-DH approaches. The MAX is under 0.60 eV for the PBE-QIDH, DSD-PBEP86, and RS-PBE-P86 functionals, while it is somewhat higher for the remainders. For the DSD-PBEP86 and PBE-QIDH methods, the MAX belongs to a Rydberg excitation, while it is affiliated to a valence excitation for the others. The picture somewhat changes for the triplet transitions. The highest overall MEs were attained by the B2GPPLYP and CIS(D) methods, while the superiors are the (SCS-)RS-PBE-P86, PBE0-2, and CCSD approaches. The error cancellation between the valence and Rydberg transitions is less relevant in these cases compared to the singlet results. The lowest overall MAX, precisely, 0.19 eV, can be observed in the case of the RS-PBE-P86 approach. Concerning the functionals, the MEs are well-balanced for the valence excitations. The highest value, 0.10 eV, was obtained by SOS-RS-PBE-P86, but this is still acceptable. The lowest MAX is around 0.20 eV obtained by the CCSD, (SCS-)RS-PBE-P86, and PBE-based approaches. The best performers regarding the MEs for the Rydberg transitions are the PBE-based, the CCSD, and the spin-scaled RS-DH methods. Significant differences between the valence and Rydberg results can only be found for the B2GPPLYP and DSD-PBEP86 functionals.

**Table 2:** Additional Error Measures for the Calculated Excitation Energies (in eV) for the Gordon Test Set^72,75^^a^

	singlet excitations						triplet excitations					
	all (63)		valence (32)		Rydberg (31)		all (38)		valence (28)		Rydberg (10)	
method	ME	MAX	ME	MAX	ME	MAX	ME	MAX	ME	MAX	ME	MAX
CCSD^b^	0.10	0.49	0.17	0.49	0.04	0.14	0.02	0.19	0.02	0.19	0.03	0.17
CC2	–0.11	0.64	–0.01	0.53	–0.22	0.64	–0.03	0.60	0.06	0.57	–0.29	0.60
CIS(D)	–0.01	1.04	0.11	1.04	–0.15	0.57	0.10	0.45	0.20	0.45	–0.19	0.45
DSD-PBEP86	–0.04	0.59	0.08	0.39	–0.18	0.59	–0.07	0.41	–0.01	0.33	–0.24	0.41
RS-PBE-P86	0.04	0.60	0.13	0.60	–0.05	0.44	–0.01	0.19	0.01	0.19	–0.08	0.18
SCS-RS-PBE-P86	0.06	0.62	0.14	0.62	–0.02	0.51	0.02	0.22	0.04	0.22	–0.04	0.14
SOS-RS-PBE-P86	0.09	0.66	0.14	0.66	0.04	0.63	0.08	0.31	0.10	0.31	0.02	0.19
B2GPPLYP^b^	–0.12	0.71	0.06	0.59	–0.32	0.71	–0.13	0.52	–0.05	0.41	–0.37	0.52
ωB2GPPLYP^b^	0.13	0.73	0.26	0.73	–0.01	0.57	–0.03	0.30	0.01	0.29	–0.14	0.30
PBE0-2	0.12	0.64	0.20	0.64	0.02	0.55	0.02	0.24	0.04	0.24	–0.04	0.14
PBE-QIDH^b^	0.13	0.52	0.22	0.49	0.02	0.52	–0.04	0.22	–0.03	0.22	–0.04	0.13

a Number of the transitions can be found in parentheses.

b Values were taken from ref (72).

On the basis of these numerical experiences, we can conclude that the overall performance of the PBE-based nonempirical DH functionals is outstanding; however, they are not recommended for singlet valence excitations. In general, the PBE0-2 method is better for valence transitions, while PBE-QIDH is more suitable for Rydberg excitations. The DSD-PBEP86 approach is the clear superior for valence transitions; however, its error is significantly higher for Rydberg excitations. This is also true for the B2GPPLYP functional, but the difference is more unbalanced. The LR-corrected ωB2GPPLYP is better in this regard; however, its performance is not outstanding in any respect. One of the most robust alternatives is the RS-PBE-P86 approach, which is an adequate choice in any case. It provides better results for singlet valence excitations compared to the PBE-based methods, while it preserves their accuracy for Rydberg excitations. Concerning the triplet transitions, its MAE is below 0.10 eV for both valence and Rydberg excitations. The SCS variant consistently improves the results, while the SOS variant can be a cost-efficient alternative concerning the relation of the accuracy and computational time. The lack of the SS contribution could be more problematic in the case of Rydberg excitations.

### 3.2 Cross-Validation

**Figure 3:**
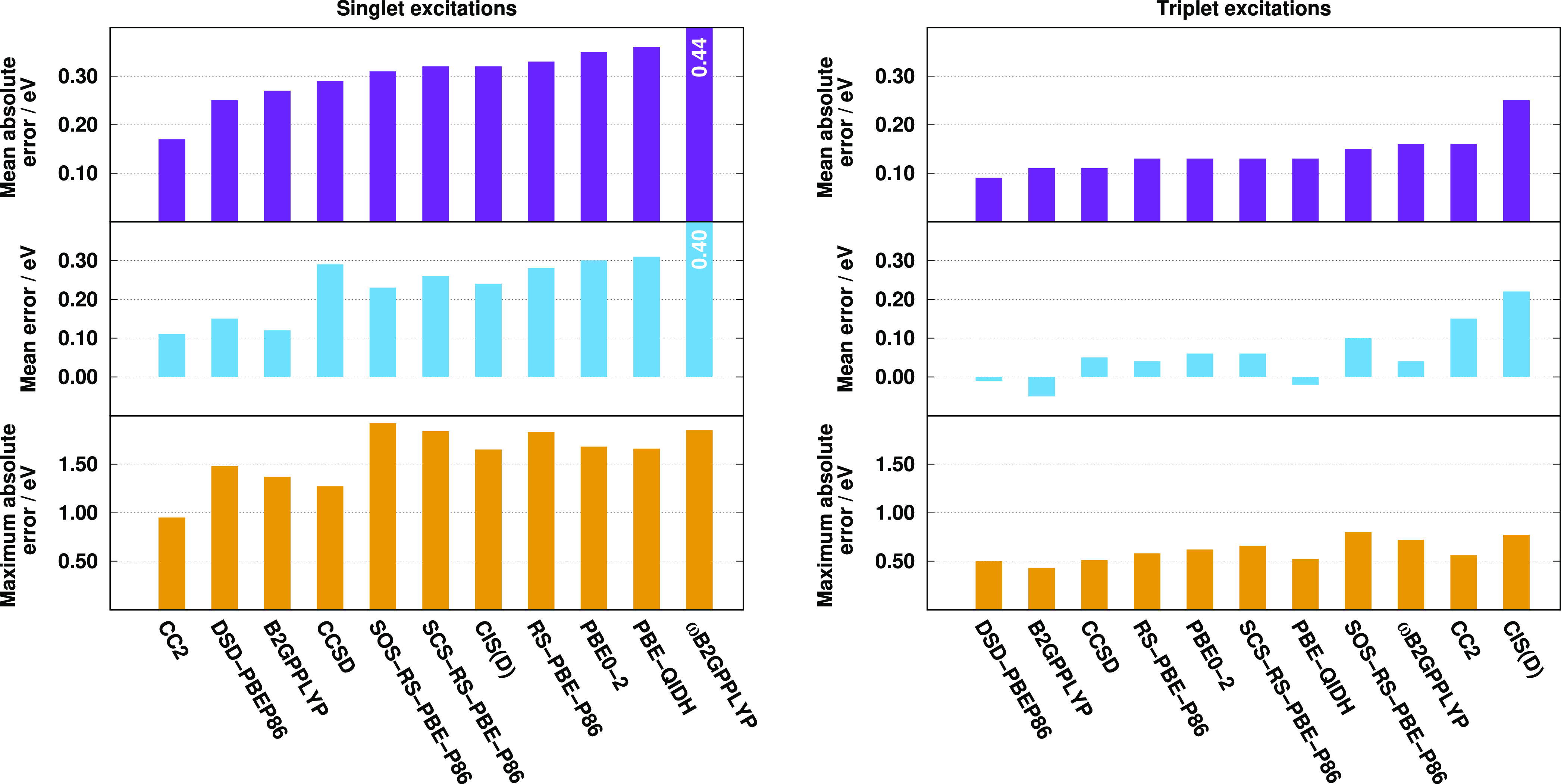
Error measures for the calculated singlet (left panel) and triplet (right panel) excitation energies for the Thiel test set^77^ using the TZVP basis sets with the def2-QZVPP-RI(-JK) auxiliary bases. The singlet (triplet) compilation contains 121 (71) transitions. The singlet and triplet CCSD values were taken from refs (113) and (77), respectively.

Cross-validation has been performed using different benchmark sets to verify the robustness of the spin-scaled variants with the default parameters determined. First, we compare the methods on the Thiel test set for this purpose. The obtained error measures are visualized in Figure 3. This benchmark set only contains valence excitations, and we already have some insights into performance of the approaches for these transitions through the Gordon test set. Again, for the singlet valence excitations, the best performers are the CC2, DSD-PBEP86, and B2GPPLYP methods with a MAE of 0.17, 0.25, and 0.27 eV, respectively. The MEs are somewhat more balanced, being around 0.12 eV for these superior models. The MAE is also below 0.30 eV for the CCSD method and starts to increase slowly for the others. The RS-DH approaches provide lower MAEs than the PBE-based ones, while the RMSEs and SDs are more favorable for the latter. The ωB2GPPLYP functional is not recommended in any respect. The MEs and MAXs are well balanced for the acceptable functionals. The SOS and SCS variants of our approach are more accurate than the unscaled one. Again, the MAEs for the triplet excitations are significantly lower. The same two functionals, namely, the DSD-PBEP86 and B2GPPLYP, are the most accurate ones with MAEs of around 0.10 eV. At the same time, interestingly, the CC2 method is one of the inferiors despite its good performance for the singlet transitions. The performances of the (SCS-)RS-PBE-P86 and PBE-based functionals are indistinguishable with a MAE of 0.13 eV, while the SOS-RS-PBE-P86 approach has a somewhat higher error, precisely, 0.15 eV. The MAE is acceptable for ωB2GPPLYP as well. The ME and MAX values are also consistent for all the methods, and salient functionals cannot be identified. The triplet excitation energies are slightly overestimated, except for the DSD-PBEP86, B2GPPLYP, and PBE-QIDH approaches, while the MAXs are around 0.50 eV.

**Figure 4:**
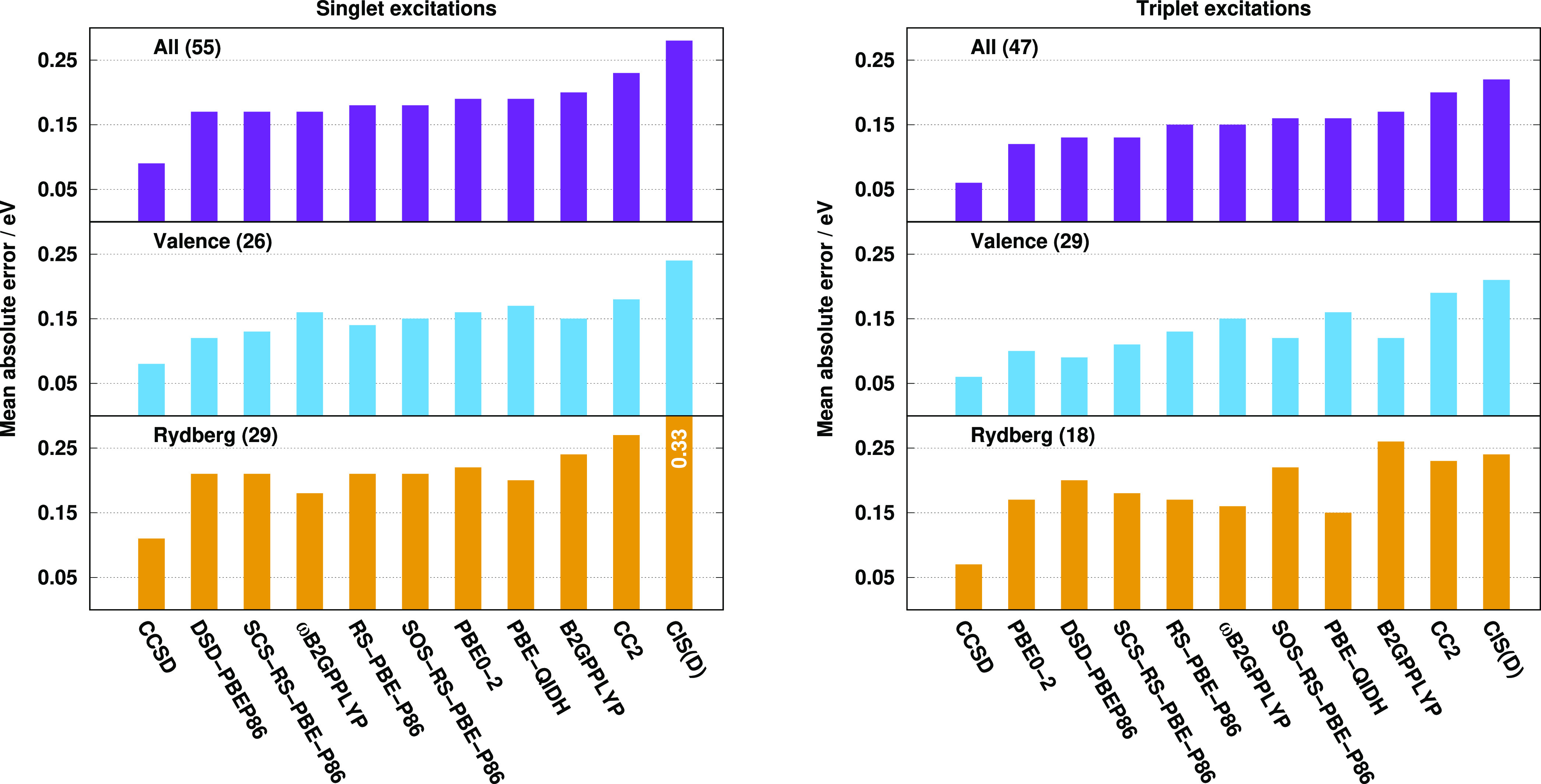
MAEs for the calculated singlet (left panel) and triplet (right panel) excitation energies for the LJ1 test set^114^ using the aug-cc-pVTZ basis sets with the corresponding auxiliary bases. The number of the transitions can be found in parentheses. The CCSD values were taken from ref (114).

Next, we assess the LJ1 test set. The results are collected in Figure 4. Inspecting the MAEs for the singlet excitations, we can conclude that the overall errors are well balanced for all the functionals. The best performers are the DSD-PBEP86, SCS-RS-PBE-P86, and ωB2GPPLYP approaches with a MAE of 0.17 eV, while the error is under 0.20 eV for the others. The inferiors are the fifth-order scaling wave-function-based methods. The outstanding accuracy of the CCSD method for valence excitations and the acceptable performance of DSD-PBEP86 for Rydberg transitions are fairly surprising. For valence excitations, the DSD-PBEP86 and (SCS-)RS-PBE-P86 approaches are recommended, while their performances for Rydberg excitations are similar with a MAE of 0.21 eV. The lowest MAE for these transitions is 0.18 eV, yielded by the ωB2GPPLYP functionals, while the highest error is only 0.24 eV obtained by the B2GPPLYP approach. Similar to the previous test sets, the MAEs for the triplet transitions are lower, however, the decrease is somewhat smaller in this case. The best performer is the PBE0-2 method, while the DSD-PBEP86 and SCS-RS-PBE-P86 functionals are also outstanding. Since the test set is dominated by valence excitations, this is not surprising. The MAE is below 0.15 eV for the RS-PBE-P86 and ωB2GPPLYP approaches as well, while it does not exceed 0.17 eV for B2GPPLYP, which is the inferior among the density functional approximations. Surprisingly, for the most accurate approaches, the MAEs for the Rydberg excitations are consistently higher than for the valence excitations. An opposite finding was observed in the case of the Gordon test set. For the former excitations, the superiors are the DSD-PBEP86, PBE0-2, and SCS-RS-PBE-P86 approaches with a MAE of around 0.10 eV, while the PBE-QIDH, ωB2GPPLYP, and RS-PBE-P86 functionals are recommended for the latter ones with a MAE of around 0.16 eV.

The compilations of the further statistical error measures for the LJ1 set can be found in Table 3. As it can be seen, the lowest overall ME for the singlet excitations is achieved by the CC2 method; however, a significant error cancellation is present between the valence and Rydberg states. It is also there but less notable for the DSD-PBEP86 and ωB2GPPLYP functionals with highly acceptable MEs. The error is still below 0.10 eV for the RS-DH approaches, while it is somewhat higher for the remainders. The same order can be determined among the functionals concerning the MAX errors. In general, the MEs and MAXs are higher for the Rydberg states compared to the valence transitions as it was so for the MAEs. Inspecting the overall MEs for the triplet excitations, we can conclude that the best performers are the CCSD, (SCS-)RS-PBE-P86, and PBE0-2 methods; however, the MEs obtained by the TDA-TDDFT approaches have an opposite sign for the two kinds of excitations. In general, the MEs are somewhat higher for the Rydberg excitations, while the MAXs belong to a valence transition. Concerning the RMSEs and SDs, for the singlet excitations, the most consistent results are obtained by the ωB2GPPLYP, DSD-PBEP86, and (SCS-)RS-PBE-P86 functionals, while DSD-PBEP86, PBE0-2, and (SCS-)RS-PBE-P86 are the superior approaches for the triplet transitions in this regard.

**Table 3:** Additional Error Measures for the Calculated Excitation Energies (in eV) for the LJ1 Set^114^^a^

	singlet excitations						triplet excitations					
	all (55)		valence (26)		Rydberg (29)		all (47)		valence (29)		Rydberg (18)	
method	ME	MAX	ME	MAX	ME	MAX	ME	MAX	ME	MAX	ME	MAX
CCSD^b^	0.09	0.38	0.08	0.19	0.10	0.38	0.02	0.36	0.00	0.36	0.04	0.26
CC2	–0.01	0.66	0.18	0.57	–0.18	0.66	0.06	0.64	0.19	0.61	–0.14	0.64
CIS(D)	0.04	0.84	0.13	0.78	–0.03	0.84	0.12	0.61	0.21	0.61	–0.03	0.53
DSD-PBEP86	–0.03	0.49	0.06	0.39	–0.11	0.49	–0.06	0.48	–0.03	0.48	–0.11	0.46
RS-PBE-P86	0.08	0.66	0.05	0.49	0.12	0.66	–0.02	0.66	–0.08	0.66	0.07	0.52
SCS-RS-PBE-P86	0.09	0.63	0.06	0.51	0.12	0.63	0.02	0.72	–0.03	0.72	0.10	0.54
SOS-RS-PBE-P86	0.10	0.58	0.08	0.57	0.11	0.58	0.09	0.82	0.05	0.82	0.15	0.58
B2GPPLYP	–0.11	0.58	–0.01	0.52	–0.20	0.58	–0.14	0.56	–0.09	0.37	–0.21	0.56
ωB2GPPLYP	0.05	0.46	0.05	0.46	0.05	0.44	–0.07	0.77	–0.10	0.77	–0.02	0.34
PBE0-2	0.14	0.77	0.13	0.35	0.14	0.77	0.02	0.73	–0.02	0.73	0.09	0.48
PBE-QIDH	0.13	0.61	0.10	0.50	0.16	0.61	–0.04	0.78	–0.11	0.78	0.08	0.40

a Number of the transitions can be found in parentheses.

b Values were taken from ref (114).

**Figure 5:**
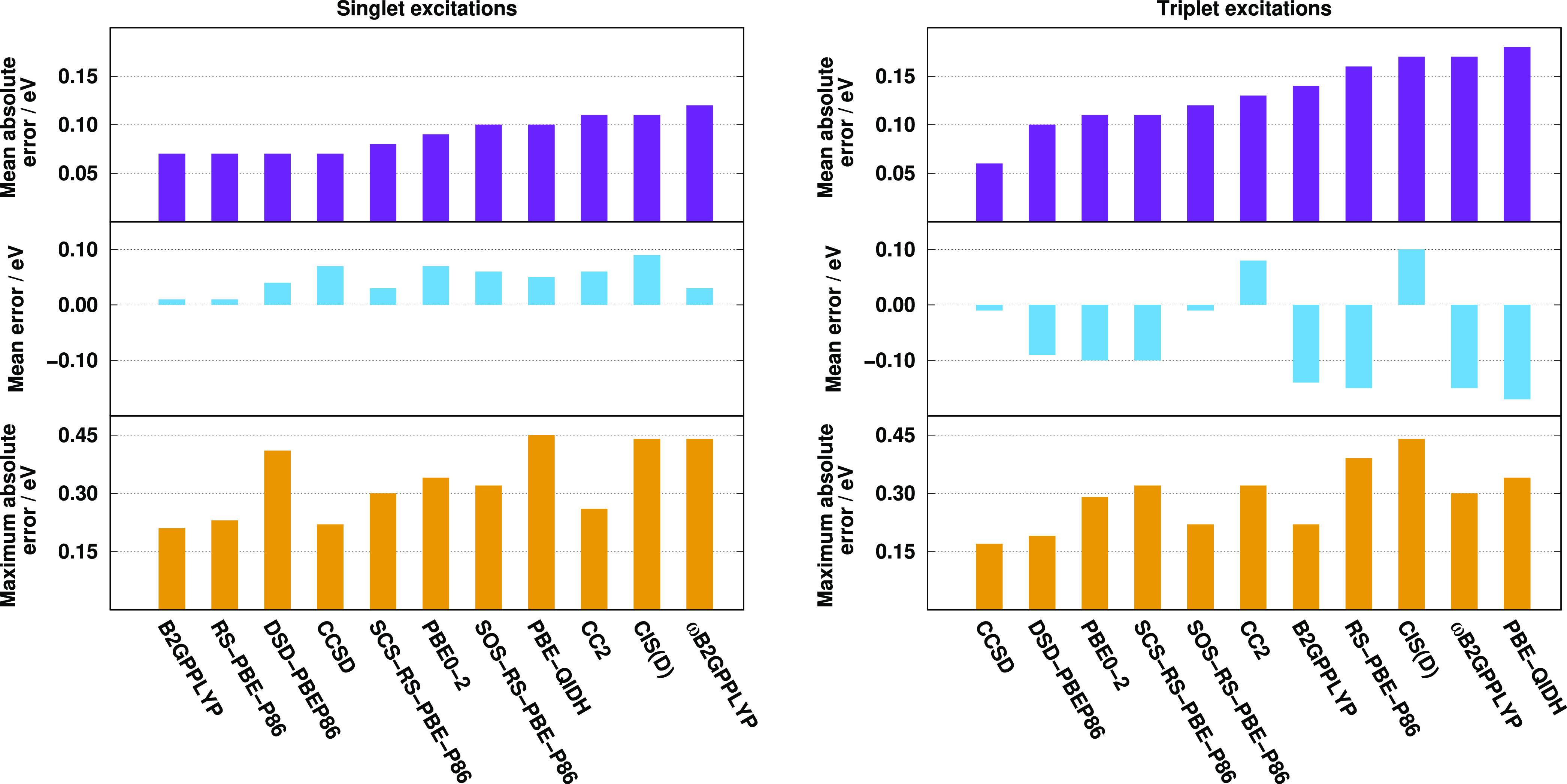
Error measures for the calculated singlet (left panel) and triplet (right panel) excitation energies for the LJ2 test set^115^ using the aug-cc-pVTZ basis sets with the corresponding auxiliary bases. The singlet (triplet) compilation contains 19 (11) transitions. The CCSD values were taken from ref (115).

Next, the second benchmark compilation of Loos, Jacquemin, and co-workers (LJ2) is inspected, which consists of the so-called “exotic” molecules. The error measures are shown in Figure 5. Since the highest MAE for the singlet excitations is only 0.12 eV, we can conclude that all the methods provide accurate results. The best performers are the B2GPPLYP, RS-PBE-P86, DSD-PBEP86, and CCSD methods with a MAE of 0.07 eV, while the error is still below 0.10 eV for the spin-scaled variants of our method and the PBE-based functionals. The MEs, which do not exceed 0.10 eV for all the methods, are well-balanced, and the excitation energies are somewhat overestimated. The MAX is under 0.30 eV for the best approaches, except for DSD-PBEP86, while it is around 0.45 eV for the less satisfactory functionals. It is interesting to see that the MAEs for the triplet excitations are significantly worse compared to the singlet results, except for CCSD. The DSD-PBEP86 approach is the winner as the MAE is 0.10 eV, while the PBE0-2 and the SCS variant of our method are also recommended with a MAE of 0.11 eV. In contrast to the singlet excitations, the MEs are systematically negative for the DHs. An almost perfect ME is attained by the SOS-RS-PBE-P86 approach, and the aforementioned methods have acceptable errors as well. The ωB2GPPLYP and PBE-QIDH approaches are inferior since their MAE and ME exceed 0.17 and 0.15 eV, respectively. The lowest MAX of around 0.20 eV is achieved by the CCSD, DSD-PBEP86, SOS-RS-PBE-P86, and B2GPPLYP methods.

**Figure 6:**
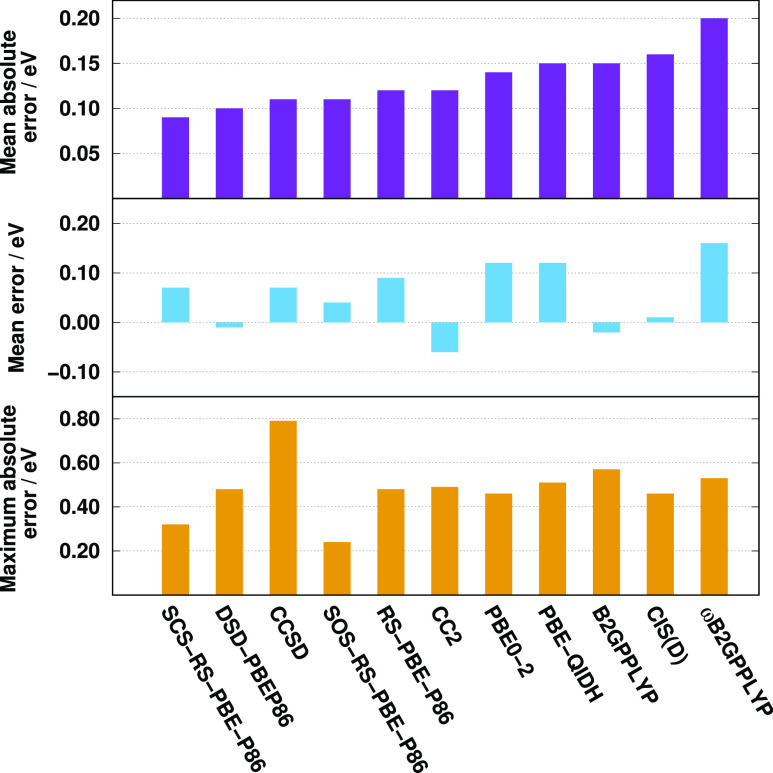
Error measures for the calculated singlet excitation energies for the SG test set^68^ using the aug-cc-pVTZ basis sets with the corresponding auxiliary bases. The B2GPPLYP, ωB2GPPLYP, PBE-QIDH, and CCSD values were taken from ref (72).

The valence excitation dominated SG test, which contains only singlet transitions, is also considered. The main error measures are visualized in Figure 6. As it can be seen, SCS-RS-PBE-P86 is superior to the other approaches with a MAE of 0.09 eV. The error is slightly higher for the DSD-PBEP86 and CCSD methods, while the performance is still excellent for the (SOS-)RS-PBE-P86 and CC2 approaches. The MAEs start to increase from this point. As it has been already shown, the PBE-based functionals and the LR-corrected ωB2GPPLYP approach are not suitable for valence excitations; however, the poor performance of B2GPPLYP is somewhat surprising. The MAE is around 0.15 eV for the genuine DHs, while it is 0.20 eV for the LR-corrected DH functional. The ME is almost perfect for the DSD-PBEP86, B2GPPLYP, and CIS(D) methods. However, only DSD-PBEP86 is recommended among them because of its relatively small MAE. The CC2 method and the spin-scaled variants of our approach are also outstanding. The lowest maximum error is obtained for SOS-RS-PBE-P86 with a MAX of 0.24 eV, while the CCSD method is inferior in this regard with a MAX of 0.79 eV in this case. The MAX errors, apart from the aforementioned approach, are very close to each other and acceptable. The lowest RMSEs and SDs are attained by the spin-scaled RS DHs.

**Figure 7:**
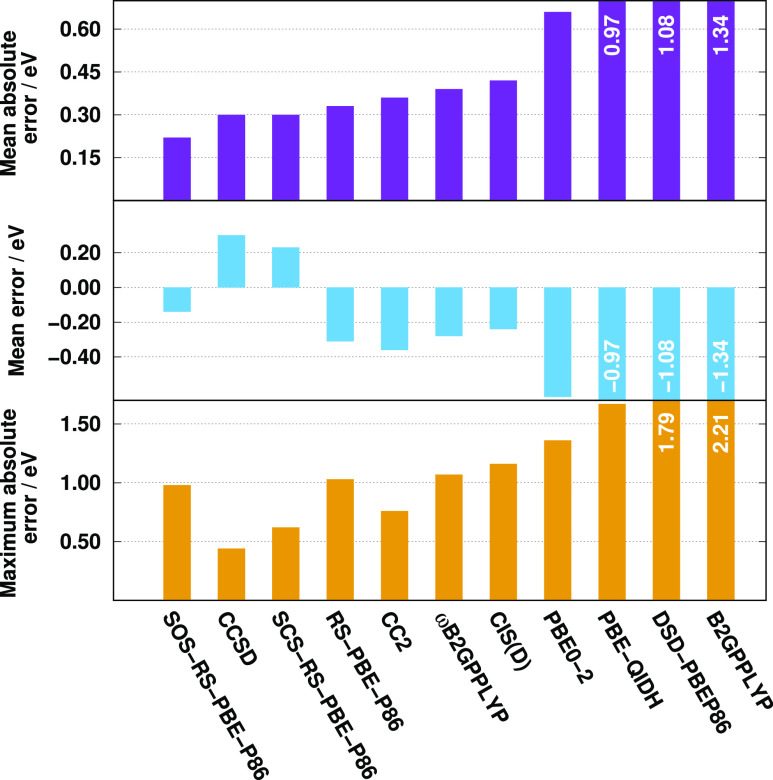
Error measures for the calculated singlet excitation energies for the CT test set^81^ using the cc-pVDZ basis sets with the corresponding auxiliary bases. The CCSD values were taken from ref (81).

Next, we study CT excitations, which present a well-known problem even in this class of methods.^71,117,118^ The numerical results for the CT benchmark set of Szalay et al. are presented in Figure 7. Inspecting the errors, the advantages of the range separation become clear since only these functionals can provide acceptable results compared to the wave-function-based methods. The lowest MAE, 0.22 eV, is attained by the SOS-RS-PBE-P86 functional, which is even better than the CCSD results. The SCS and unscaled variants are also superior to CC2, while the ωB2GPPLYP has excellent results as well. The error is around 0.30 eV for the former RS DHs, while it is 0.39 eV for the LR-corrected DH functional. The standard DHs are highly not recommended. The MAE is barely tolerable, 0.66 eV, for the PBE0-2 functional but is around 1.00 eV for the PBE-QIDH and DSD-PBEP86 and even worse for the B2GPPLYP functional. This order does not change when the MEs are considered. The errors are outstanding for the RS and LR-corrected DHs, while PBE0-2 is the best standard DH functional. It is interesting to see that the CCSD and SCS-RS-PBE-P86 methods overestimate the excitation energies, whereas they are systematically underestimated for the others. The lowest MAXs are produced by the former two methods, while it is around 1.00 eV for the (SOS-)RS-PBE-P86 and ωB2GPPLYP functionals. This relatively large number is still outstanding as the maximum error is at least 1.36 eV but could be more than 2.00 eV for the standard DHs. The RMSE for CCSD and the spin-scaled RS DHs is 0.31 and 0.34 eV, respectively, while it is under 0.50 eV for the CC2 and RS-PBE-P86 methods. Excellent SDs were achieved by CCSD, CC2, and SCS-RS-PBE-P86. Interestingly, the DSD-PBEP86 and PBE-QIDH functionals provide better results in this measure than ωB2GPPLYP.

**Figure 8:**
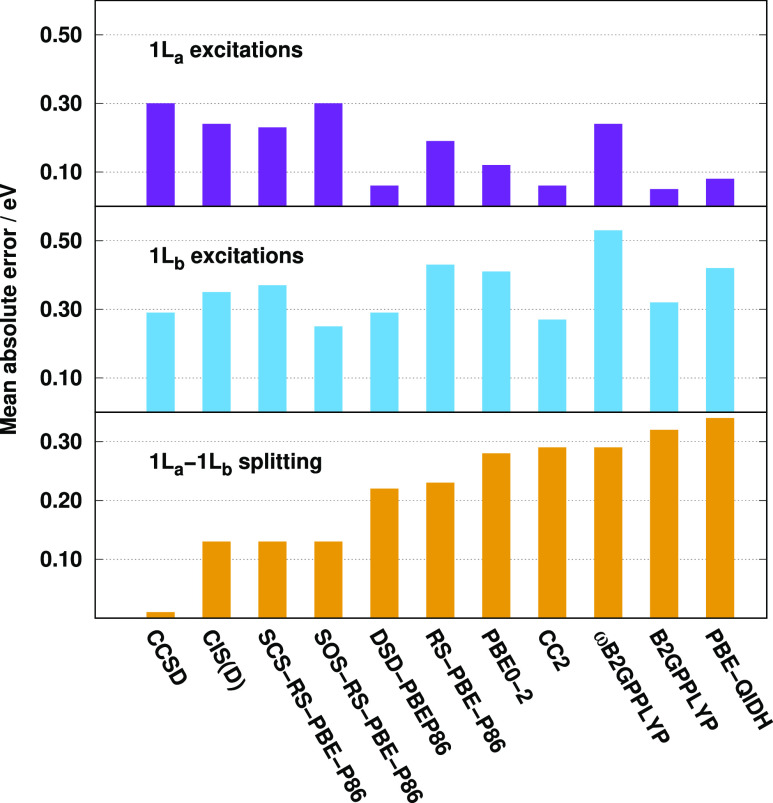
MAEs for the calculated lowest two excitations for the linear PAHs and their splitting^79^ using the cc-pVTZ basis sets with the corresponding auxiliary bases. The CCSD values were taken from ref (79).

The last benchmark set for the cross-validation tests the lowest two singlet excitations and their splitting for the linear PAHs from naphthalene to hexacene. The MAEs are visualized in Figure 8. Inspecting the bars for the ^1^L_a_ excitations, we can observe that the results are fairly hectic. The lowest MAEs can be obtained by the B2GPPLYP, DSD-PBEP86, CC2, and PBE-QIDH methods. The errors are around 0.06 eV in these cases. For PBE0-2, it is somewhat higher, while the MAEs are around 0.24 eV for the RS and LR-corrected functionals. Interestingly, one of the worst results, 0.30 eV, is attained by the CCSD method. Almost the same order can be determined for the ^1^L_b_ excitations; however, the MAEs are noticeably higher, and the differences between the performances are less significant. One of the most notable changes is that the spin-scaled RS-DH approaches are competitive for these excitations. That is, the lowest MAE, 0.25 eV, is obtained by the SOS-RS-PBE-P86 functional, while it is around 0.30 eV for several methods as well. The splitting of the lowest two excitations is also an important measure for PAHs. In this respect, the most remarkable results are surprisingly produced by the CCSD method with an almost perfect MAE. The CIS(D) and the spin-scaled RS-DH models are also outstanding with a MAE of 0.13 eV. The performances of the DSD-PBEP86 and RS-PBE-P86 approaches are also acceptable. For the rest of the functionals, the error is noticeably higher; however, they do not seem to be less reliable than the CC2 method, where the MAE is 0.29 eV.

## 4 Conclusions

The major contribution of this study is twofold. First, our recently presented two-parameter RS-DH TDA-TDDFT approach^70^ has been combined with spin-scaling techniques. The proposed SCS variant provides higher flexibility to the energy functional, while the SOS variant could be a cost-efficient alternative concerning the relation of the accuracy and computational time. Second, considering the facts that almost 500 excitations are assessed and a wide range of phenomena are studied, this work is one of the most comprehensive studies in the excited-state DH theory. All in all, 320 singlet and 167 triplet excitations are inspected relying on the broadly used benchmark sets or the most recently proposed ones. In addition, challenging excitations, which present a well-known problem even in this class of methods, are also assessed.

The spin-scaling factors of the ansatz were determined using the singlet excitations of the well-balanced Gordon test set. Thereafter, cross-validation was performed on several benchmark sets using the default parameters. Our numerical results show that the DSD-PBEP86 method has an outstanding accuracy for valence transitions; however, its error is significantly higher for Rydberg excitations. The overall performances of the PBE-based nonempirical DH functionals are well balanced, and they are superior to the empirical B2GPPLYP approach. Among the former functionals, PBE0-2 is more suitable for valence transitions, while PBE-QIDH is more reliable for Rydberg excitations; however, they are not recommended for singlet valence excitations in general. The LR-corrected ωB2GPPLYP approach provides a more robust alternative compared to the B2GPPLYP functional; however, its results are not outstanding in any respect. One of the most robust performances is attained by the RS-PBE-P86 approach, which is an adequate choice in any case. The SCS variant consistently improves the results, while the SOS variant preserves the benefits of the original RS-DH method decreasing its costs at the same time. The necessity of the range separation was demonstrated for CT excitations, as it was also pointed out in refs (71)(117), and (118). Finally, on average, the errors are lower for the triplet excitations in most cases; however, this effect is not trivial.

## Supporting Information Available

Computed excitation energies (XLSX)
